# Supplementary material for: A new conceptual framework for the transformation of groundwater dissolved organic matter
Source: Nat Commun. 2022 Apr 20;13:2153. doi: 10.1038/s41467-022-29711-9 (PMC9021313; doi:10.1038/s41467-022-29711-9)
Supplement: Supplementary file 1 — Supplementary Information [file 41467_2022_29711_MOESM1_ESM.pdf]

Supplementary Information

A new conceptual framework for the transformation of groundwater dissolved organic matter

Liza K. McDonough[1,2*], Martin S. Andersen[2,3], Megan I. Behnke[4], Helen Rutlidge[2,3], Phetdala Oudone[2,5], Karina Meredith[1], Denis M. O'Carroll[2,3], Isaac R. Santos[6], Christopher E. Marjo[7], Robert G.M. Spencer[4], Amy M. McKenna[8], Andy Baker[2,5]

[1] Australian Nuclear Science and Technology Organisation (ANSTO), New Illawarra Rd, Lucas Heights, NSW, 2234, Australia

[2] Connected Waters Initiative Research Centre, UNSW Sydney, NSW, 2052, Australia

[3] School of Civil and Environmental Engineering, UNSW Sydney, NSW, 2052, Australia

[4] Department of Earth, Ocean, and Atmospheric Science, Florida State University, Florida, 32310, USA

[5] School of Biological, Earth and Environmental Sciences, UNSW Sydney, NSW, 2052, Australia

[6] National Marine Science Centre, Southern Cross University, Coffs Harbour, NSW, 2450, Australia.

[7] Mark Wainwright Analytical Centre, UNSW Sydney, NSW 2052, Sydney, Australia

[8] National High Magnetic Field Laboratory, Florida State University, Tallahassee, Florida 32310-4005, USA

*Corresponding author: lizam@ansto.gov.au

Contents:

Supplementary Figure 1 – Supplementary Figure 7

Supplementary Table 1 – Supplementary Table 5

Supplementary Note 1

Supplementary References

Supplementary Figures

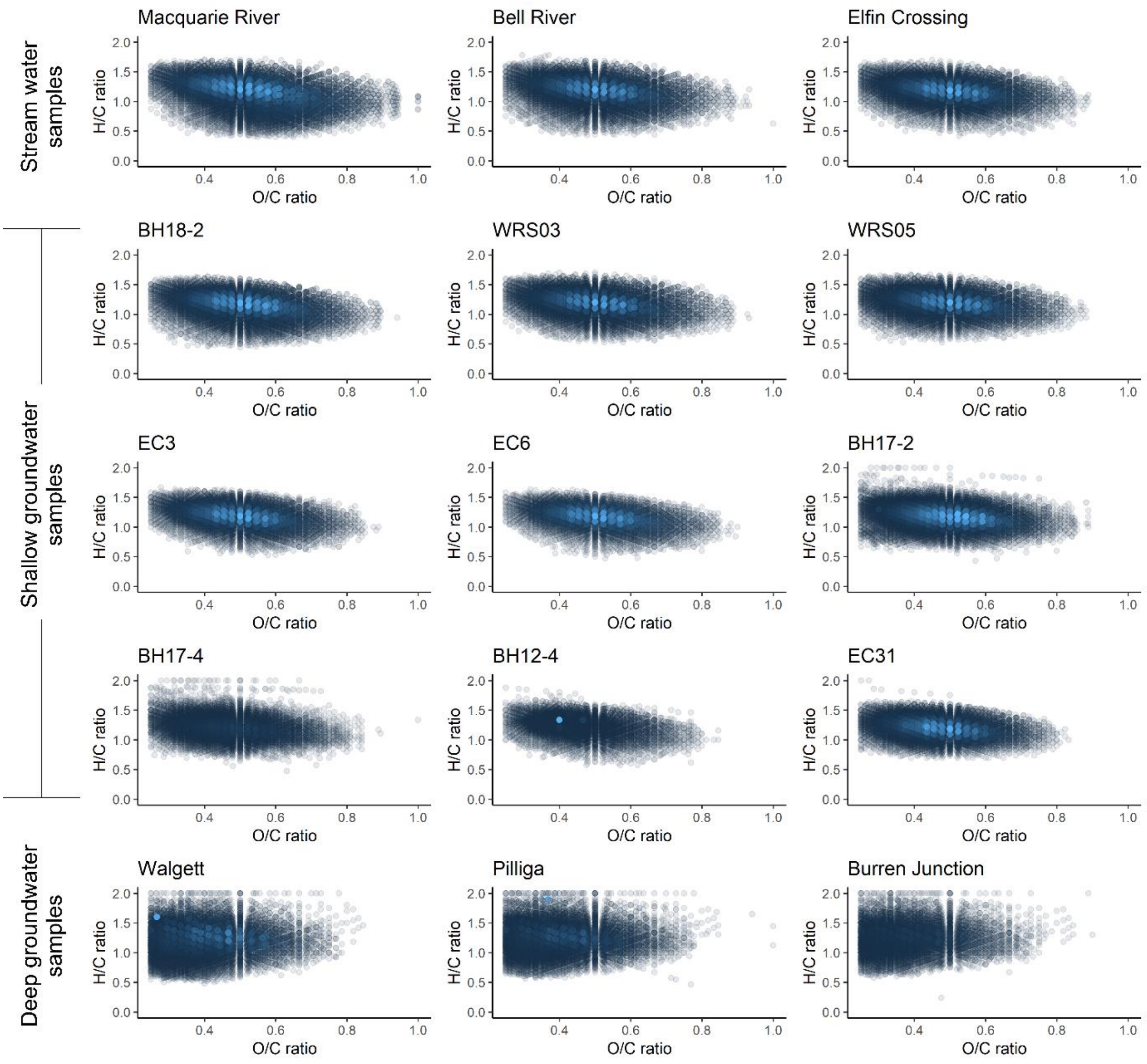

Supplementary Figure 1. Van Krevelen diagrams for each sample. Formulae coloured light blue with greater opacity represent those with higher relative intensity whilst formulae in dark blue with greater transparency represent those with lower intensity relative to the rest of the formulae in the sample. Samples are shown in order of DOC age with youngest to oldest samples displayed from left to right, top to bottom in each group (stream water, shallow groundwater samples and deep groundwater samples).

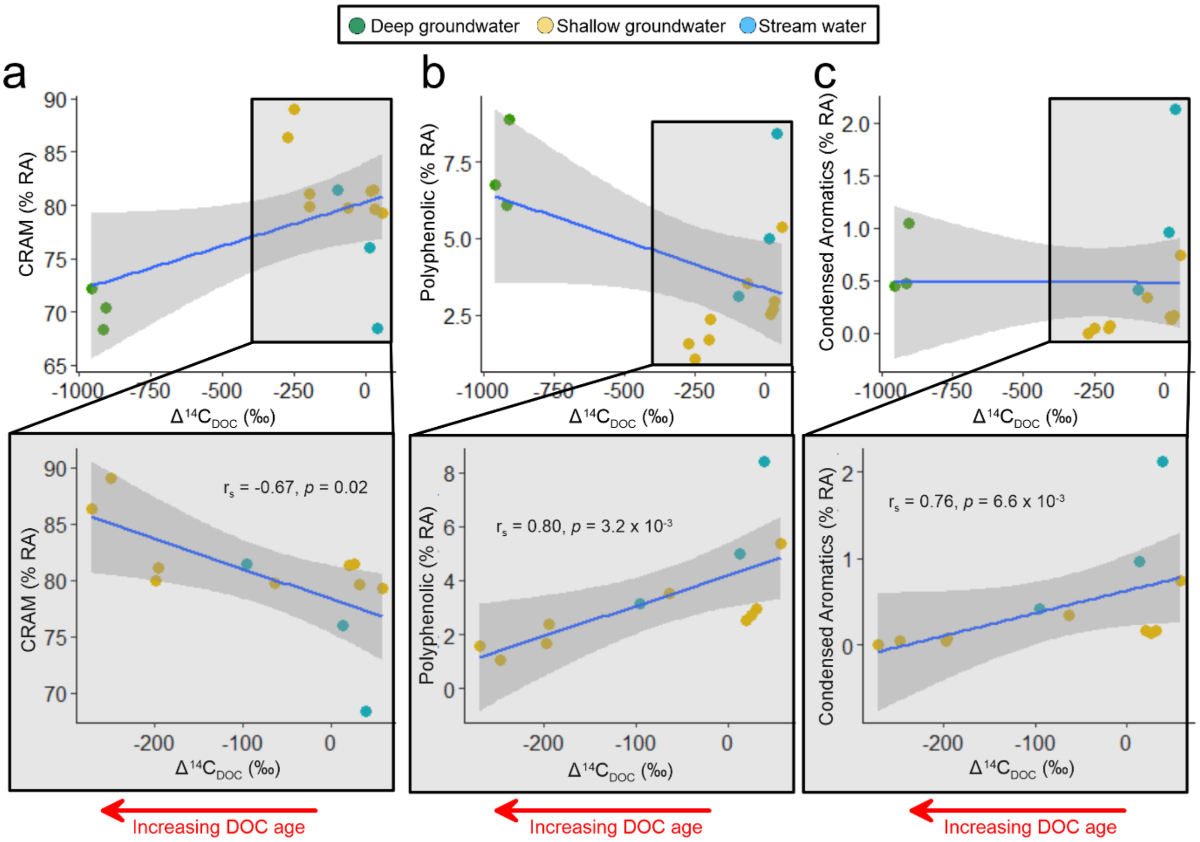

Supplementary Figure 2. Spearman's correlations between $\Delta^{14}C_{DOC}$ (‰) and a) weighted sample average % RA of carboxylic-rich alicyclic molecules (CRAM), b) weighted sample average % RA of polyphenolic formulae, and c) weighted sample average % RA of condensed aromatic formulae. $\Delta^{14}C_{DOC}$ represents the per mille (‰) depletion or enrichment of ^{14}C relative to the standard, normalised for isotope fractionation [1]. Lower $\Delta^{14}C_{DOC}$ (‰) values to the left of the x-axis represent lower ^{14}C content and more highly aged DOC samples as indicated by the red arrows. Blue, yellow and green symbols in the main plots of a-c represent stream water, shallow groundwater, and deep groundwater respectively. Grey shaded area represents the 95% confidence interval on the regression line shown in blue. Spearman correlations (r_s) and associated *p*-values are shown. These graphs highlight the significant decline in % RA of polyphenolics and condensed aromatics with decreasing $\Delta^{14}C_{DOC}$ (‰), and the significant increase in % RA CRAM with decreasing $\Delta^{14}C_{DOC}$ (‰) in surface and shallow groundwaters only.

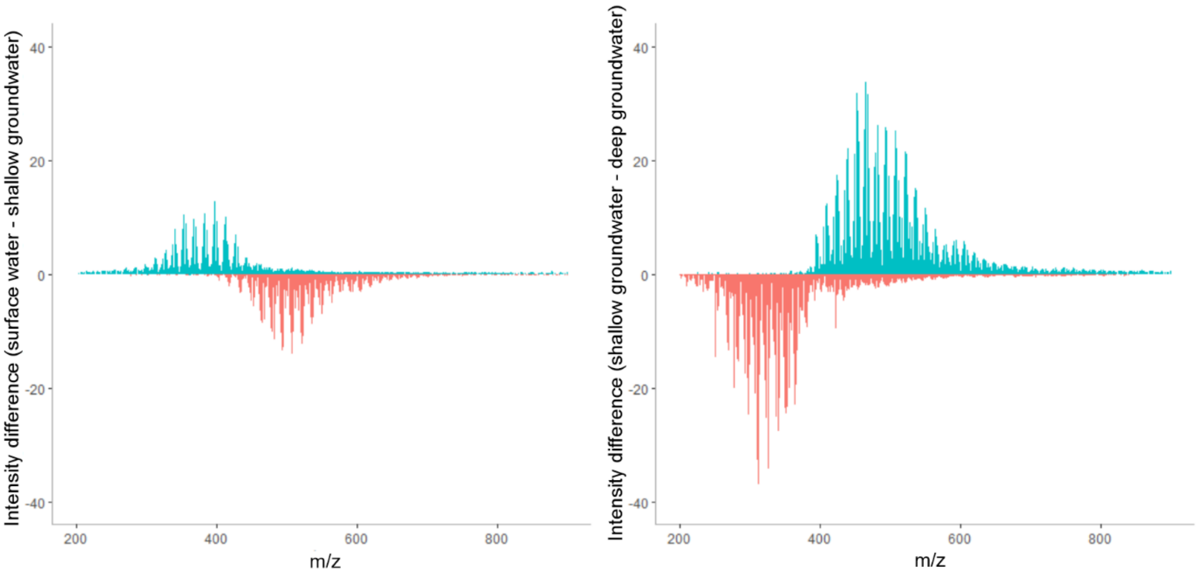

Supplementary Figure 3: Bar plots showing the mass associated with formulae higher (blue) and lower (red) in intensity in surface water compared to shallow groundwater (left), and formulae higher (blue) and lower (red) in intensity in shallow groundwater compared to deep groundwater.

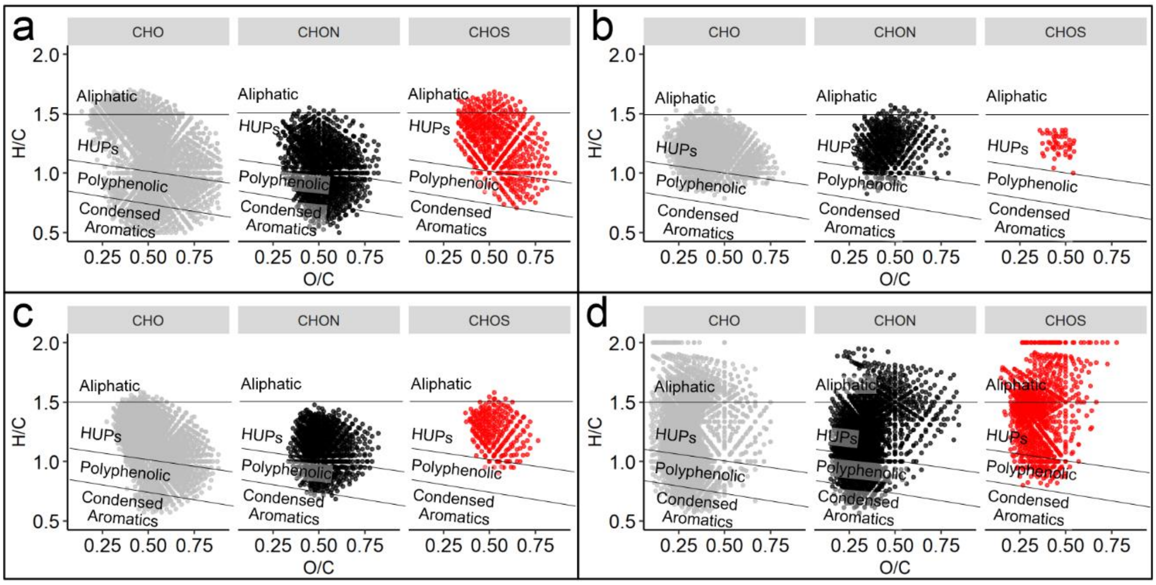

Supplementary Figure 4: van Krevelen Diagrams for each class (CHO, CHON or CHOS) of DOM formulae a) higher in median relative intensity (intensity difference < 0) in stream water compared to shallow groundwater, b) higher in median relative intensity (intensity difference < 0) in shallow groundwater compared to stream water, c) higher in median relative intensity (intensity difference < 0) in shallow groundwater compared to deep groundwater, and d) higher in median relative intensity (intensity difference < 0) in deep groundwater compared to shallow groundwater (see also Supplementary Table 5). Point colours in the VKD's correspond to CHO (grey), CHON (red) and CHOS (black) formulae. Categories of compounds (aliphatic, highly unsaturated and phenolic (HUPs), polyphenolic and condensed aromatics) are separated by solid black lines. Note: the median differences in molecule intensities have been used in the VKDs to reduce the influence of outliers with very high or low compound intensity. Larger numbers and range of H/C and O/C are observed for heteroatom-containing (CHON and CHOS) formulae in stream water and deep groundwater (a and d) compared to shallow groundwater (b and c) where the ranges are confined to relatively intermediate values predominantly within the HUPs category. Deep groundwaters show a pronounced increase in deoxygenated compounds (d). Notably, no CHOS formulae are found in the condensed aromatic category.

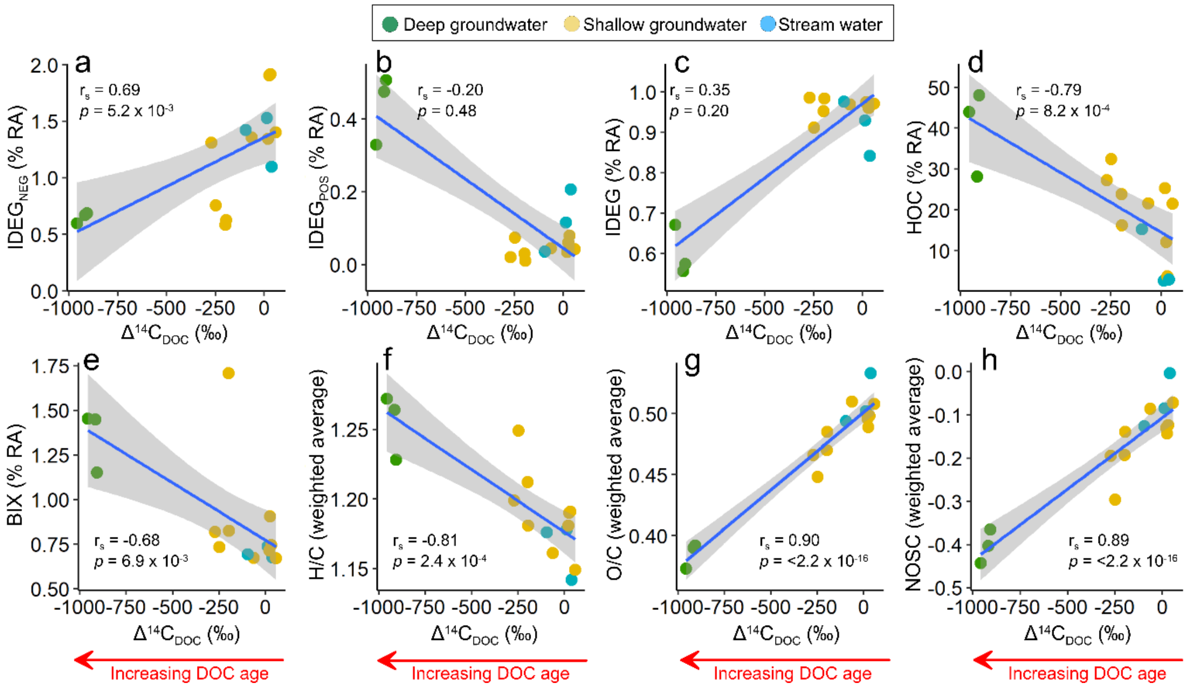

Supplementary Figure 5: Correlations of DOM parameters with $\Delta^{14}C_{DOC}$ (‰). $\Delta^{14}C_{DOC}$ represents the per mille (‰) depletion or enrichment of ^{14}C relative to the standard, normalised for isotope fractionation [1]. Lower $\Delta^{14}C_{DOC}$ (‰) values to the left of the x-axis represent lower ^{14}C content and more highly aged DOC samples as indicated by the red arrows. Plots show the relationship between $\Delta^{14}C_{DOC}$ (‰) and: (a) percent relative abundance (% RA) of $IDEG_{NEG}$, b) $IDEG_{POS}$ and c) IDEG calculated per Flerus, et al. [2], d) % RA hydrophobic organic carbon (HOC), e) BIX, f) weighted average H/C ratios, g) weighted average O/C ratios, and h) weighted average nominal oxidation state of carbon (NOSC). Blue, yellow and green symbols in the main plots of a-c represent stream water, shallow groundwater, and deep groundwater respectively. Regression lines are shown as blue lines, with grey shading representing the 95% confidence interval around the line. Spearman correlations (r_s) and corresponding p-values are shown. HOC is included here since the higher relative electronegativity of O (3.44) compared to C (2.55) [3] makes high O/C formula more polar and hydrophilic [4], and consequently more biodegradable [5]. The removal of these high O/C formulae in groundwater over time results in a negative correlation between the percent relative abundance (% RA) of hydrophobic DOM and $\Delta^{14}C_{DOC}$ (‰) (p = 8.55 x 10^{-4}).

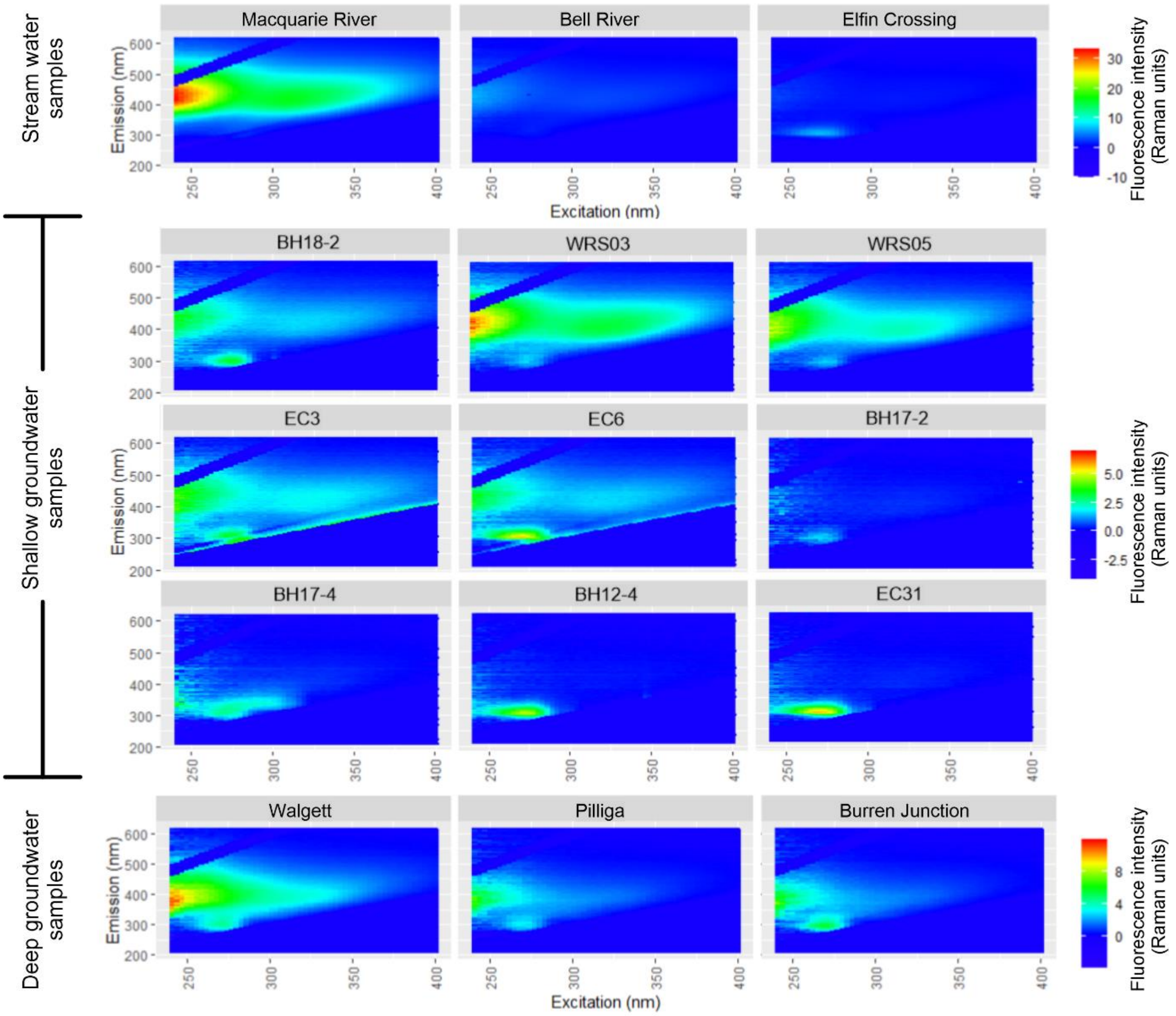

Supplementary Figure 6. Excitation emission matrices (EEMs) for each sample. Samples are shown in order of DOC age with youngest to oldest samples displayed from left to right, top to bottom in each group (stream water, shallow groundwater samples and deep groundwater samples).

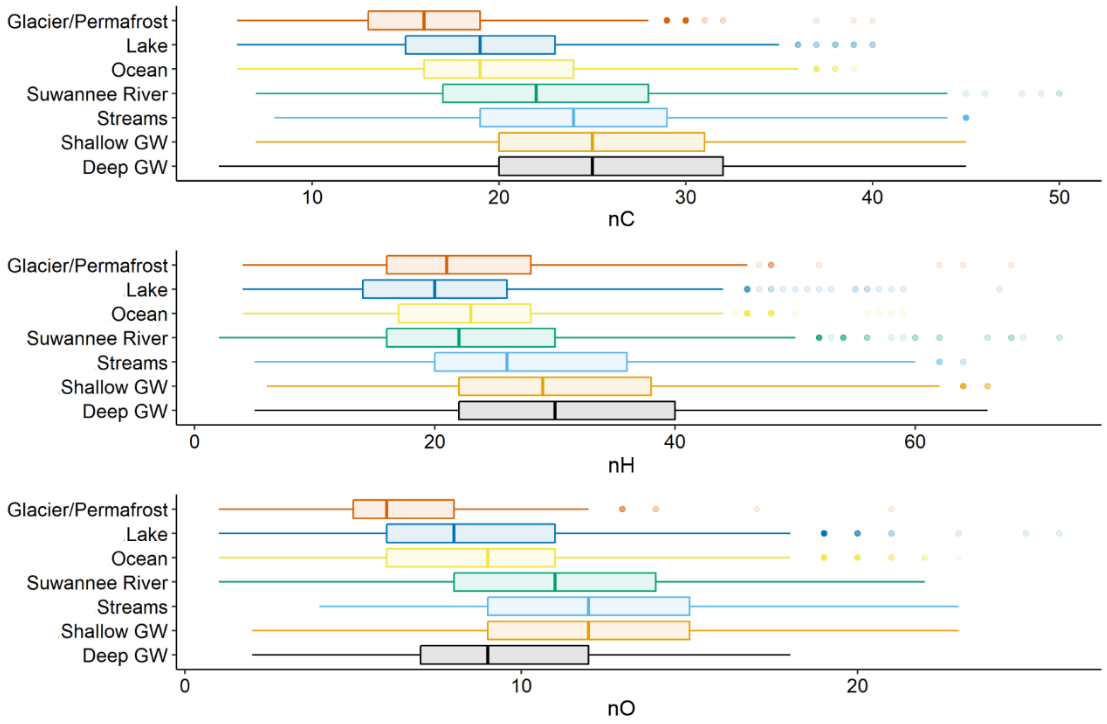

Supplementary Figure 7. Boxplots showing numbers of C (nC), O (nO) and H (nH) per formula in various environments. Boxes represent the interquartile range, containing median (solid vertical line), with whiskers representing the upper and lower 25% of data. Circles represent outliers. "Glacier/permafrost" refers to formulae (n = 4,028) identified in an Antarctic supraglacial stream [6] and Arctic permafrost [7], "Lake" referes to formulae (n = 16,696) identified in Swedish [8] and German [9] lakes, "Ocean" refers to formulae (n = 25,376) identified in the Mediterranean Sea [9,10], North Sea [9,11], Antarctic bottom water [9] and North Atlantic deep water [9], "Suwannee River" refers to formulae (n = 7,745) identified in IHSS Suwannee River reference sample [6], "Streams" refers to all formulae (n = 10,661) identified in Macquarie River, Bell River and Elfin Crossing samples from this study, "Shallow GW" refers to formulae (n = 12,742) identified in shallow groundwater samples from this study and "Deep GW" refers to deep groundwater samples from this study (n formulae = 11,811).

Supplementary Tables

Supplementary Table 1. Screen depths in meters below ground surface (m bgs) and redox state (per McMahon and Chapelle [12]) of samples assessed in this study from Maules Creek, NSW, Wellington, NSW and from the Great Artesian Basin in Walgett, Pilliga and Burren Junction, NSW.

Sample	Screen depth (m bgs)	DO (mg/l)	N-NO_3^- (mg/l)	Mn^{2+} (mg/l)	Fe^{2+} (mg/l)	SO_4^{2-} (mg/l)	Redox category [12]
				**Maules Creek**			
**BH17-2 (MC)**	10.0	6.16	1.12	<0.01	<0.01	3.12	Oxic
**BH17-4 (MC)**	35.97	5.85	0.92	0.03	0.04	2.94	Oxic
**Elfin Crossing (MC)**	Surface water	7.25	<0.01	0.04	0.03	6.53	Oxic
**BH18-2 (MC)**	11.50	0.27	2.12	0.00	0.00	2.87	NO_3^- reducing
**EC6 (MC)**	4.55	0.18	<0.01	1.58	1.05	8.53	Fe (III) / SO_4^{2-} reducing
**EC3 (MC)**	3.11	0.16	<0.01	1.37	1.78	7.03	Fe (III) / SO_4^{2-} reducing
**EC31 (MC)**	12.32	0.19	<0.01	0.01	<0.01	19.96	Suboxic
**BH12-4 (MC)**	40.70	0.17	<0.01	0.16	<0.01	18.03	Mn (IV) reducing
				**Great Artesian Basin**			
**Pilliga**	> 500	0.13	<0.01	<0.01	0.06	0.08	Methanogenic
**Burren Junction**	> 500	0.02	<0.01	<0.01	0.10	0.10	Methanogenic
**Walgett**	> 500	0.02	<0.01	<0.01	0.03	0.26	Methanogenic
				**Wellington**			
**Bell River (W)**	Surface water	9.41	0.51	0.02	0.03	53.8	Oxic
**Macquarie River (W)**	Surface water	9.23	0.17	0.02	0.04	17.8	Oxic
**WRS03 (W)**	9.01	0.27	<0.01	0.13	0.13	36.0	Fe (III) / SO_4^{2-} reducing
**WRS05 (W)**	18.08	1.71	0.05	<0.01	0.01	79.5	Oxic

Supplementary Table 2. Liquid chromatography-organic carbon detection, Fourier-transform ion cyclotron resonance mass spectrometry (FT-ICR MS) and fluorescence results. Samples are shown in order of DOC age with youngest to oldest samples displayed from left to right, top to bottom in each group (stream water, shallow groundwater samples and deep groundwater samples). $Mass_w$, H/C_w, O/C_w, N/C_w, $NOSC_w$, $AImod_w$ and DBE_w refer to the intensity weighted average molecular mass, hydrogen/carbon ratio, oxygen/carbon ratio, nitrogen/carbon ratio, nominal oxidation state, modified aromaticity index and double bond equivalents respectively. DOM groups obtained from liquid chromatography organic carbon detection (LC-OCD) including hydrophobic organic carbon (HOC), chromatographable dissolved organic carbon (CDOC), biopolymers (BP), humics and low molecular weight neutrals (LMWN) are shown as percent relative abundance (% RA). Fourier-transform ion cyclotron resonance mass spectrometry (FT-ICR MS) DOM groups including island of stability (IOS) formulae and carboxylic-rich alicyclic molecules (CRAM) are also shown as percent relative abundance (% RA).

	**Stream Water**			**Shallow Groundwater**									**Deep Groundwater**		
Sample ID	Macquarie River	Bell River	Elfin Crossing Surface	BH18-2	WRS03	WRS05	EC3	EC6	BH17-2	BH 17-4	BH 12-4	EC31	Pilliga	Burren Junction	Walgett
**LC-OCD**															
DOC (mg/L)	8.26	1.87	1.34	1.24	2.07	1.58	1.12	1.16	0.69	0.48	0.68	0.75	0.75	1.1	0.79
HOC (% RA)	3	3	15	21	4	12	25	22	16	24	32	27	28	44	48
CDOC (% RA)	97	97	85	79	96	88	75	79	84	76	68	73	72	56	52
BP (% RA)	2.1	5.5	7.2	1.2	0.2	0.4	1.1	0	3.4	0.6	0.7	0	2	0.6	0.9
Humics (% RA)	65	60	45	44	67	53	45	46	39	34	13	24	22	16	24
BB (% RA)	19	16	7	17	8	19	11	14	11	6	25	11	11	5	8
LMWN (% RA)	12	16	25	17	21	16	19	19	31	36	29	38	38	35	19
Humic substance molecular weight (g mol^{-1})*	581	269	119	675	192	325	201	257	338	285	113	110	370	241	260
**FT-ICR MS**															
Molecular formulae (#)	5401	4247	5927	7444	8989	7451	5645	5220	9418	6758	5220	7444	9948	7304	9869
$Mass_w$	476	465	497	494	472	470	502	497	528	506	460	492	428	400	418
HC_w	1.14	1.18	1.18	1.15	1.19	1.19	1.18	1.16	1.18	1.21	1.25	1.2	1.26	1.27	1.23
OC_w	0.53	0.5	0.49	0.51	0.5	0.49	0.5	0.51	0.49	0.47	0.45	0.47	0.39	0.37	0.39
NC_w	0.006	0.012	0.005	0.005	0.006	0.007	0.005	0.004	0.009	0.011	0.003	0.009	0.007	0.008	0.007
$NOSC_w$	-0.004	-0.085	-0.126	-0.072	-0.124	-0.143	-0.130	-0.086	-0.139	-0.193	-0.296	-0.195	-0.403	-0.443	-0.365
$AImod_w$	0.32	0.30	0.30	0.32	0.30	0.30	0.30	0.31	0.30	0.29	0.29	0.30	0.30	0.31	0.33

DBE_w	10.6	10.2	10.9	11.1	10.2	10.3	10.9	10.9	11.6	10.9	9.7	10.8	8.9	9.4	9.7
CHO (%RA)	85.6	79.6	88.6	89.5	85.5	86.2	89.4	92.3	82.7	79.1	93.5	85.0	78.3	77.3	78.4
CHON (%RA)	10.2	15.9	9.1	8.8	9.2	10.7	9.1	7.7	13.9	17.4	5.1	12.4	13.9	14.5	14.2
CHOS (%RA)	4.2	4.3	2.3	1.8	4.8	3.1	1.5	0.0	3.4	3.6	1.5	2.3	7.8	8.2	7.4
CHONS (%RA)	0.00	0.18	0.00	0.00	0.43	0.00	0.00	0.00	0.00	0.00	0.00	0.28	0.00	0.00	0.00
Sugars (%RA)	0.25	0.02	0.00	0.00	0.00	0.00	0.00	0.00	0.00	0.00	0.00	0.00	0.01	0.01	0.01
Peptide-like (%RA)	0.1	0.1	0.0	0.0	0.1	0.1	0.1	0.1	0.3	1.0	0.1	0.1	1.4	2.0	1.2
Condensed aromatics (%RA)	2.1	1.0	0.4	0.8	0.2	0.1	0.2	0.3	0.1	0.1	0.0	0.0	0.5	0.5	1.1
Polyphenolic (%RA)	8.4	5.0	3.1	5.4	3.0	2.7	2.5	3.5	2.4	1.7	1.1	1.6	6.1	6.8	8.9
Highly unsaturated & phenolic (%RA)	85.8	90.9	94.8	92.7	94.6	95.2	95.9	95.0	95.1	93.5	96.6	96.8	80.1	77.2	78.4
Aliphatic (%RA)	3.4	3.0	1.7	1.2	2.2	1.9	1.3	1.0	2.1	3.8	2.2	1.6	12.0	13.6	10.5
I_{DEG}	0.84	0.93	0.98	0.97	0.96	0.97	0.97	0.97	0.98	0.95	0.91	0.99	0.56	0.67	0.57
IOS (% RA)	27.1	31.9	34.3	33.4	36.1	37.2	34.3	35.7	29.2	27.1	25.6	32.5	20.5	19.3	20.0
CRAM (% RA)	68.4	76.0	81.4	79.2	79.6	81.5	81.3	79.7	81.1	79.9	89.0	86.3	68.4	72.2	70.4
**Fluorescence**															
Fluorescence Index	1.29	1.31	1.36	1.38	1.35	1.48	1.28	1.41	1.41	1.23	0.86	1.25	1.74	1.81	1.76
Biological Index	0.67	0.74	0.69	0.67	0.74	0.9	0.71	0.67	0.83	1.71	0.73	0.82	1.45	1.45	1.15
Peak B	2.29	1.29	5.96	3.24	1.49	1.39	3.19	4.79	1.44	2.42	4.43	5.15	2.91	4.05	3.97
Peak T	4.29	1.44	2.1	1.3	1.37	1.03	1.6	1.68	0.65	2.01	1.35	1.46	2.23	2.84	3.7
Peak A	25.67	4.87	2.15	2.72	4.79	3.72	3.06	2.69	0.86	1.27	0.71	0.91	4.15	4.09	8.06
Peak M	16.38	3.03	1.42	1.64	3.22	2.36	1.92	1.65	0.48	0.81	0.33	0.44	1.94	1.83	3.83
Peak C	11.92	2.44	1.17	1.43	2.7	1.87	1.72	1.45	0.37	0.41	0.28	0.31	0.96	0.96	2.36

*Humic substance molecular weight values are calibrated using surface waters (e.g., oceans, rivers, and lakes) which contain humic substance molecular weights of >350 g mol^{-1} (Huber et al., 2011). Values for groundwater samples containing low humic substance molecular weights (<350 g mol^{-1}) may therefore be unreliable.

Supplementary Table 3. ^{14}C and ^{13}C dissolved organic carbon (DOC) and dissolved inorganic carbon (DIC) results. NDFB denotes a $^{14}C_{DIC}$ value that is not detectable from the background (i.e., highly aged with very low ^{14}C content). Conventional radiocarbon DOC and DIC ages are rounded according to Stuiver and Polach [1]. Values are listed as “modern” where the sample is calculated as being younger than the radiocarbon reference year of 1950 [1]. $\Delta^{14}C_{DOC}$ (‰) values used in figures and in the main text were calculated from International Atomic Energy Agency [13] using the pMC values displayed below.

Sample ID	$^{14}C_{DOC}$ (pMC)	Conventional radiocarbon DOC age (yBP) ± 1 σ	$\delta^{13}C_{DOC}$ (‰)	$^{14}C_{DIC}$ (pMC)	Conventional radiocarbon DIC age (yBP) ± 1 σ	$\delta^{13}C_{DIC}$ (‰)
Pilliga	8.30 ± 0.44	20,000 ± 440	-12.3 ± 0.5	0.06 ± 0.03	>54,100	-7.5 ± 0.3
Burren Junction	4.28 ± 0.32	25,310 ± 600	-25.5 ± 0.5	0.08 ± 0.04	>51,100	-6.7 ± 0.3
Walgett	9.30 ± 0.38	19,080 ± 330	-23.9 ± 0.5	0.02 ± 0.02	NDFB	-7.3 ± 0.3
BH17-2	80.44 ± 0.44	1,750 ± 45	-25.3 ± 0.5	103.10 ± 0.32	Modern	-16.9 ± 0.3
BH17-4	80.21 ± 0.30	1,770 ± 30	-25.6 ± 0.5	97.22 ± 0.31	225 ± 30	-16.4 ± 0.3
WRS03	103.14 ± 0.33	Modern	-25.7 ± 0.4	101.71 ± 0.22	Modern	-16.8 ± 0.3
WRS05	102.59 ± 0.86	Modern	-24.7 ± 0.4	96.64 ± 0.22	275 ± 20	-16.3 ± 0.3
BH12-4	75.22 ± 0.35	2,285 ± 40	-24.0 ± 0.5	61.91 ± 0.14	3,850 ± 20	-13.5 ± 0.3
BH18-2	105.77 ± 0.56	Modern	-25.5 ± 0.5	93.72 ± 0.22	520 ± 20	-14.0 ± 0.3
EC31	72.91 ± 0.34	2,540 ± 40	-24.2 ± 0.5	108.07 ± 0.20	Modern	-14.7 ± 0.3
EC3	102.04 ± 0.37	Modern	-36.1 ± 0.4	104.2 ± 0.21	Modern	-16.1 ± 0.3
EC6	93.64 ± 0.30	530 ± 30	-25.6 ± 0.5	103.66 ± 0.2	Modern	-16.0 ± 0.3
Bell River	101.32 ± 0.38	Modern	-26.0 ± 0.4	97.77 ± 0.22	180 ± 20	-10.0 ± 0.3
Macquarie River	103.88 ± 0.28	Modern	-26.4 ± 0.4	102.92 ± 0.24	Modern	-10.0 ± 0.3
Elfin Crossing Surface	90.44 ± 0.28	805 ± 25	-26.6 ± 0.5	104.29 ± 0.22	Modern	-13.3 ± 0.3

Supplementary Table 4. Intensity weighted percent relative abundance (% RA) of compound groups and classes of molecules unique to stream water samples (n = 1,084), shallow groundwater samples (n = 1,921), deep groundwater DOM samples (n = 4,735), and formulae present in all 15 samples (n = 2,137). CHO CHON CHOS and CHONS compounds refers to the presence of carbon (C), hydrogen (H), oxygen (O), nitrogen (N) and sulfur (S) atoms in the elemental compositions. Compounds with a heteroatom refers to formulae containing C, H, O and at least one N or S atom.

	Unique to stream water DOM (weighted % RA)	Unique to shallow groundwater DOM (weighted % RA)	Unique to deep GW DOM (weighted % RA)	Common to all samples (weighted % RA)
**Compound Category**				
Highly unsaturated and phenolic (low O/C)	2.6	43.2	49.0	56.5
Highly unsaturated and phenolic (high O/C)	35.9	41.3	1.0	40.1
Peptide-like	0.1	5.7	7.6	0.0
Aliphatic (low O/C)	1.2	2.0	15.2	1.5
Aliphatic (high O/C)	2.1	0.7	1.4	0.2
Condensed aromatics with a heteroatom	12.6	0.3	2.5	0.0
Condensed aromatics without a heteroatom (CHO)	11.3	1.2	1.6	0.0
Polyphenolic (High O/C)	2.0	4.7	21.7	1.3
Polyphenolic (Low O/C)	25.5	0.8	0.0	0.5
Sugars with a heteroatom	0.2	0.0	0.0	0.0
Sugars without a heteroatom (CHO)	6.6	0.0	0.0	0.0
Mass	482.2	585.6	444.2	461.8
DBE	13.0	12.2	11.4	10.0
AI_{mod}	0.47	0.23	0.35	0.30
NOSC	0.61	-0.05	-0.50	-0.21
**Class**				
CHO	54.1	23.9	38.7	94.1
CHON	33.5	57.4	44.4	5.9
CHOS	12.2	12.0	16.9	0.0
CHONS	0.2	6.6	0.0	0.0

Supplementary Table 5. Percent relative abundance of compound groups and classes of molecules higher in median intensity in stream water vs shallow groundwater, shallow groundwater vs stream water, shallow groundwater vs deep groundwater and deep groundwater vs shallow groundwater. The median relative intensity of each molecule in stream water was subtracted from the median relative concentration of each formula in shallow groundwater. Formulae with a result > 0 were deemed to be higher in median concentration in stream water compared to shallow groundwater, whilst formulae with a result < 0 were deemed to be higher in median concentration in shallow groundwater compared to stream water. Similarly, the median relative intensity of each formula in shallow groundwater water was subtracted from the median relative concentration of each formula in the deep groundwater samples. Formulae with a result > 0 were deemed to be higher in shallow groundwater compared to deep groundwater, whilst formulae with a result < 0 were deemed to be higher in median concentration in deep groundwater compared to shallow groundwater. CHO CHON CHOS and CHONS compounds refers to the presence of carbon (C), hydrogen (H), oxygen (O), nitrogen (N) and sulfur (S) atoms in the elemental compositions. Compounds with a heteroatom refers to molecules containing C, H, O and at least one N or S atom.

	Higher in stream water vs shallow groundwater (weighted %RA)	Higher in shallow groundwater vs stream water (weighted %RA)	Higher in shallow groundwater vs deep groundwater (weighted %RA)	Higher in deep groundwater vs shallow groundwater (weighted %RA)
**Compound Category**				
Aliphatic (high O/C)	3.1	0.0	0.1	1.5
Aliphatic (low O/C)	4.8	0.1	0.1	15.6
Highly unsaturated and phenolic (high O/C)	56.1	42.2	63.6	11.0
Highly unsaturated and phenolic (low O/C)	19.0	57.3	34.2	58.6
Condensed aromatics without a heteroatom (CHO)	3.4	0.0	0.1	0.6
Condensed aromatics with a heteroatom (CHON, CHOS or CHONS)	0.8	0.0	0.0	0.3
Polyphenolic (High O/C)	9.6	0.0	1.5	0.2
Polyphenolic (Low O/C)	3.0	0.4	0.3	9.8
Peptide-like	0.1	0.1	0.0	2.4
Sugars without a heteroatom (CHO)	0.1	0.0	0.0	0.0
Sugars with a heteroatom (CHON, CHOS or CHONS)	0.0	0.0	0.0	0.0
**Class**				
CHO	73.6	95.9	89.2	72.6
CHON	15.8	3.9	9.1	17.3
CHOS	10.6	0.2	1.7	10.1

Supplementary Note

Supplementary Note 1. An increase in the intensities of 1,144 aromatic low O/C, low H/C formulae (mainly comprised of polyphenolics) in the deep groundwaters were observed. These aromatic formulae accounted for 10.8% (intensity weighted) of the formulae that are higher in deep than shallow groundwater (Figure 1D). Though often associated with terrestrial inputs from vascular plants, similar low O/C, low H/C formulae have also been identified in microbial metabolites and biomass from marine and supraglacial environments [14,15]. Processes including desorption from iron oxides with increasing pH [16] and/or the transfer of eletrons generated during mineralisation to non-aromatic quinoid moieties [17] could also result in the build-up of aromatic formulae including phenols [18]. As the GAB contains coal measures known as the Walloon Coal Measures, we consider the potential for aromatic and polyphenolic formulae shown in Figure 1D to result from coal inputs in the three GAB groundwaters. DOM inputs from coal are aromatic and characterised by low m/z values (100 – 300) and increasingly low H/C and O/C as coal rank increases [19]. The Walloon Coal Measures are classified as bituminous thermal black coals, which typically have higher carbon energy (lower O/C) content than lignite or sub-bituminous coals. The intensity increases of polyphenolic and condensed aromatic formulae in these three deep confined groundwater samples are low in the region on the Van Krevelen space that aligns with bituminous coals (H/C = ~ 0.5 – 1.0, O/C < 0.15) [19]. Furthermore, the five aromatic formulae showing the highest intensity increases compared to the shallow groundwater in the three deep groundwater samples ($C_{19}H_{18}O_7$, $C_{18}H_{18}O_5$, $C_{17}H_{16}O_7$, $C_{19}H_{18}O_6$ and $C_{17}H_{16}O_6$) all contain m/z values between 313 – 357. In contrast, coal tar and its distillate and residue fractions have been shown contain peaks at m/z ranges of 100 – 300 [20]. This agrees with the m/z values of the five most prominent FT-ICR mass spectra peaks identified in bituminous coal from New Zealand ($C_9H_5O_6$, $C_9H_5O_7$, $C_9H_5O_8$, $C_{10}H_5O_8$, $C_{10}H_5O_9$) [21], which all similarly contain m/z > 300. None of these five peaks were identified in the deep groundwater samples from this study, suggesting that the polypenolic and other aromatic formulae enriched in the deep groundwater samples are unlikely to originate from coal inputs. As a result, we suggest that the aromatic formulae present in the deep groundwater samples are likely sourced from microbial metabolites, biomass or desorbed material, and preserved by a lack of photodegradation and thermodynamic equilibria.

Supplementary References

1. Stuiver, M. & Polach, H. A. Discussion Reporting of 14C Data. *Radiocarbon* **19**, 355-363, doi:10.1017/S0033822200003672 (1977).
2. Flerus, R. *et al.* A molecular perspective on the ageing of marine dissolved organic matter. *Biogeosciences* **9**, 1935-1955, doi:https://doi.org/10.5194/bg-9-1935-2012 (2012).
3. Ouellette, R. J. & Rawn, J. D. in *Principles of Organic Chemistry* Ch. 1, 1-32 (Elsevier, 2015).
4. Han, L., Kaesler, J., Peng, C., Reemtsma, T. & Lechtenfeld, O. J. Online Counter Gradient LC-FT-ICR-MS Enables Detection of Highly Polar Natural Organic Matter Fractions. *Analytical Chemistry*, doi:https://doi.org/10.1021/acs.analchem.0c04426 (2020).
5. Hongve, D., Van Hees, P. A. W. & Lundström, U. S. Dissolved components in precipitation water percolated through forest litter. *European Journal of Soil Science* **51**, 667-677, doi:https://doi.org/10.1111/j.1365-2389.2000.00339.x (2000).
6. Smith, H. J. *et al.* Dynamic processing of DOM: Insight from exometabolomics, fluorescence spectroscopy, and mass spectrometry. *Limnology and Oceanography Letters* **3**, 225-235, doi:https://doi.org/10.1002/lol2.10082 (2018).
7. Spencer, R. G. M. *et al.* Detecting the signature of permafrost thaw in Arctic rivers. *Geophysical Research Letters* **42**, 2830-2835, doi:https://doi.org/10.1002/2015GL063498 (2015).
8. Kellerman, A. M., Kothawala, D. N., Dittmar, T. & Tranvik, L. J. Persistence of dissolved organic matter in lakes related to its molecular characteristics. *Nature Geoscience* **8**, 454-457, doi:https://doi.org/10.1038/ngeo2440 (2015).
9. Zark, M. & Dittmar, T. Universal molecular structures in natural dissolved organic matter. *Nature Communications* **9**, 3178, doi:https://doi.org/10.1038/s41467-018-05665-9 (2018).
10. Martínez-Pérez, A. M. *et al.* Linking optical and molecular signatures of dissolved organic matter in the Mediterranean Sea. *Scientific Reports* **7**, 3436, doi:https://doi.org/10.1038/s41598-017-03735-4 (2017).
11. Osterholz, H. *et al.* Deciphering associations between dissolved organic molecules and bacterial communities in a pelagic marine system. *The ISME Journal* **10**, 1717-1730, doi:https://doi.org/10.1038/ismej.2015.231 (2016).
12. McMahon, P. B. & Chapelle, F. H. Redox Processes and Water Quality of Selected Principal Aquifer Systems. *Groundwater* **46**, 259-271, doi:https://doi.org/10.1111/j.1745-6584.2007.00385.x (2008).
13. International Atomic Energy Agency. Isotope methods for dating old groundwater. (Vienna, Austria, 2013).
14. Lechtenfeld, O. J., Hertkorn, N., Shen, Y., Witt, M. & Benner, R. Marine sequestration of carbon in bacterial metabolites. *Nature Communications* **6**, 6711, doi:https://doi.org/10.1038/ncomms7711 (2015).
15. Antony, R. *et al.* Molecular Insights on Dissolved Organic Matter Transformation by Supraglacial Microbial Communities. *Environmental Science & Technology* **51**, 4328-4337, doi:httpa://doi.org/10.1021/acs.est.6b05780 (2017).
16. Avena, M. J. & Koopal, L. K. Desorption of Humic Acids from an Iron Oxide Surface. *Environmental Science & Technology* **32**, 2572-2577, doi:https://doi.org/10.1021/es980112e (1998).
17. Lovley, D. R., Coates, J. D., Blunt-Harris, E. L., Phillips, E. J. P. & Woodward, J. C. Humic substances as electron acceptors for microbial respiration. *Nature* **382**, 445-448, doi:https://doi/org/10.1038/382445a0 (1996).
18. Lau, M. P. & del Giorgio, P. Reactivity, fate and functional roles of dissolved organic matter in anoxic inland waters. *Biology Letters* **16**, 20190694, doi:https://doi.org/10.1098/rsbl.2019.0694 (2020).

19 Agraniotis, M., Bergins, C., Stein-Cichoszewska, M. & Kakaras, E. in *Low-Rank Coals for Power Generation, Fuel and Chemical Production* (eds Zhongyang Luo & Michalis Agraniotis) pp. 95-124 (Woodhead Publishing, 2017).

20 Shi, Q. *et al.* Identification of Dihydroxy Aromatic Compounds in a Low-Temperature Pyrolysis Coal Tar by Gas Chromatography−Mass Spectrometry (GC−MS) and Fourier Transform Ion Cyclotron Resonance Mass Spectrometry (FT-ICR MS). *Energy & Fuels* **24**, 5533-5538, doi:https://doi.org/10.1021/ef1007352 (2010).

21 Zhu, Y., Vieth-Hillebrand, A., Noah, M. & Poetz, S. Molecular characterization of extracted dissolved organic matter from New Zealand coals identified by ultrahigh resolution mass spectrometry. *International Journal of Coal Geology* **203**, 74-86, doi:https://doi.org/10.1016/j.coal.2019.01.007 (2019).
